# The ZIP6/ZIP10 heteromer is essential for the zinc-mediated trigger of mitosis

**DOI:** 10.1007/s00018-020-03616-6

**Published:** 2020-08-14

**Authors:** Thirayost Nimmanon, Silvia Ziliotto, Olivia Ogle, Anna Burt, Julia M. W. Gee, Glen K. Andrews, Pete Kille, Christer Hogstrand, Wolfgang Maret, Kathryn M. Taylor

**Affiliations:** 1 Department of Pathology, Phramongkutklao College of Medicine, 315 Ratchawithi Road, Thung Phayathai, Ratchathewi, Bangkok, 10400 Thailand; 2 Breast Cancer Molecular Pharmacology Group, School of Pharmacy and Pharmaceutical Sciences, Redwood Building, Cardiff University, King Edward VII Avenue, Cardiff, CF10 3NB UK; 3 Departments of Biochemistry and Molecular Biology, Kansas City, USA; 4 Anatomy and Cell Biology, Medical Center, University of Kansas, Kansas City, KS 66106 USA; 5 School of Biosciences, Cardiff University, Sir Martin Evans Building, Museum Avenue, Cardiff, CF10 3AT UK; 6 Metal Metabolism Group, Diabetes and Nutritional Sciences Division, Faculty of Life Sciences and Medicine, King’s College London, 150 Stamford Street, London, SE1 9NH UK

**Keywords:** SLC39A6, SLC39A10, Zinc transport, Cell division, pSer^727^STAT3, Cell cycle progression, Cell growth

## Abstract

Zinc has been known to be essential for cell division for over 40 years but the molecular pathways involved remain elusive. Cellular zinc import across biological membranes necessitates the help of zinc transporters such as the SLC39A family of ZIP transporters. We have discovered a molecular process that explains why zinc is required for cell division, involving two highly regulated zinc transporters, as a heteromer of ZIP6 and ZIP10, providing the means of cellular zinc entry at a specific time of the cell cycle that initiates a pathway resulting in the onset of mitosis. Crucially, when the zinc influx across this heteromer is blocked by ZIP6 or ZIP10 specific antibodies, there is no evidence of mitosis, confirming the requirement for zinc influx as a trigger of mitosis. The zinc that influxes into cells to trigger mitosis additionally changes the phosphorylation state of STAT3 converting it from a transcription factor to a protein that complexes with this heteromer and pS^38^Stathmin, the form allowing microtubule rearrangement as required in mitosis. This discovery now explains the specific cellular role of ZIP6 and ZIP10 and how they have special importance in the mitosis process compared to other ZIP transporter family members. This finding offers new therapeutic opportunities for inhibition of cell division in the many proliferative diseases that exist, such as cancer.

**Electronic supplementary material:**

The online version of this article (10.1007/s00018-020-03616-6) contains supplementary material, which is available to authorized users.

## Introduction

Zinc is essential for life [1] and as such has indispensable roles in most biological systems. At least 10% of the human proteome are zinc-binding proteins [2], and more than 3% of all genes encode proteins with zinc finger domains. Accordingly, zinc plays a vital role in processes that are essential for cell survival, including signal transduction, gene expression, meiosis [3], immune functions [4], control of apoptosis, and cell cycle progression [5]. Zinc is vital during different cell cycle stages [6–8] as well as indispensable for passage through G2/M [8], suggestive of a regulatory role for zinc in mitotic entry. Furthermore, zinc has been known for over 40 years to be crucial for cell division, as established by demonstration of a zinc-dependent step in the G2 stage of the cell cycle [9] and how zinc was essential for progression from G2 to mitosis [10], confirming the ability of zinc to reverse a divalent cation chelating agent induced suppression of cell cycle progression [11], although the exact molecular mechanism is still unknown.

Despite many years of study of the role of zinc in enzymes and transcription factors, it has more recently been classified as an intracellular second messenger, transducing an extracellular stimulus into intracellular fluctuations of zinc ions that affect signalling cascades [12]. One pathway involves the release of zinc from a store in the endoplasmic reticulum [13] which activates multiple downstream pathways required to promote cell survival and growth.

Due to its role in cell proliferation, zinc has been investigated in cancer for many years. Serum zinc can decrease in cancer [14–17] whereas zinc within the cancer tissue is often elevated compared to normal tissue perhaps reflecting the higher requirement for growth [18–20]. One exception to this is prostate cancer where the zinc is decreased [21]. Accordingly, chelating zinc has been explored as a means to stop proliferative growth of cancer tissues [22]. The additional zinc seen in cancers is usually supplied by various zinc transporters from the SLC39A family [23] [24–26]. Two close homologues within this family, ZIP6 (SLC39A6, also known as LIV-1) and ZIP10 (SLC39A10) [27, 28], with a 43.5% sequence identity, are on the same clade of the ZIP family phylogenetic tree [29] and have both been independently implicated in cancer [23, 29]. ZIP6 was first discovered as an oestrogen-regulated gene [30] present in breast cancers with lymph node involvement [31] and more recently used as a biomarker of oestrogen-receptor-positive luminal-type-A breast cancer [32, 33], a relationship confirmed both in cell lines and clinical material [34]. ZIP10 has also been connected to cancer progression as a marker of metastatic breast cancer [35], an indicator of aggressiveness of renal cell carcinoma [36] and associated with the expression of oestrogen receptor, ERBB3 and STAT3 in clinical breast cancer [34]. Interestingly, STAT3 is also known to increase the expression of ZIP6, a protein promoting cell migration [23, 37].

The observed similarity between ZIP6 and ZIP10 is reinforced by our recent demonstration of a ZIP6/ZIP10 heteromer [29]. This ZIP6/ZIP10 heteromer has a functional role in epithelial-mesenchymal transition (EMT), a fundamental event during gastrulation and cancer metastasis enabling individual cells to lose their cell–cell adherence, allowing cell rounding and detachment. Removal of either the ZIP10 gene [29] or the ZIP6 gene [38] produces an identical phenotype in zebrafish gastrulation, causing EMT by removal of the adherence protein *E* cadherin (CDH1).

Grouping together the fact that no mechanism exists to explain the obligatory zinc required for mitosis and that both ZIP6 and ZIP10 cause cell rounding, the first step in the mitotic pathway, we examined whether these transporters have any function in mitosis. Here we reveal how the zinc influx across the ZIP6/ZIP10 heteromer has a crucial purpose to initiate mitosis. Furthermore, we demonstrate the essential role of the ZIP6/ZIP10 heteromer in driving mitosis by the ability of our ZIP6/ZIP10 blocking antibodies to completely prevent mitotic entry, even in the presence of agents that cause mitosis. Additionally, we determine important interacting proteins in this pathway by demonstrating that the ZIP6/ZIP10 heteromer interacts with both pS^727^STAT3, the phosphorylation of which is zinc-dependant, and pS^38^Stathmin, a known regulator of mitotic entry [39]. These data together define how zinc initiates the mitotic pathway and opens a new research avenue for novel therapeutic targets for diseases whose phenotypes include increased cell proliferation, such as cancer.

## Results

### The requirement of ZIP6 and ZIP10 for mitosis

**Fig. 1:**
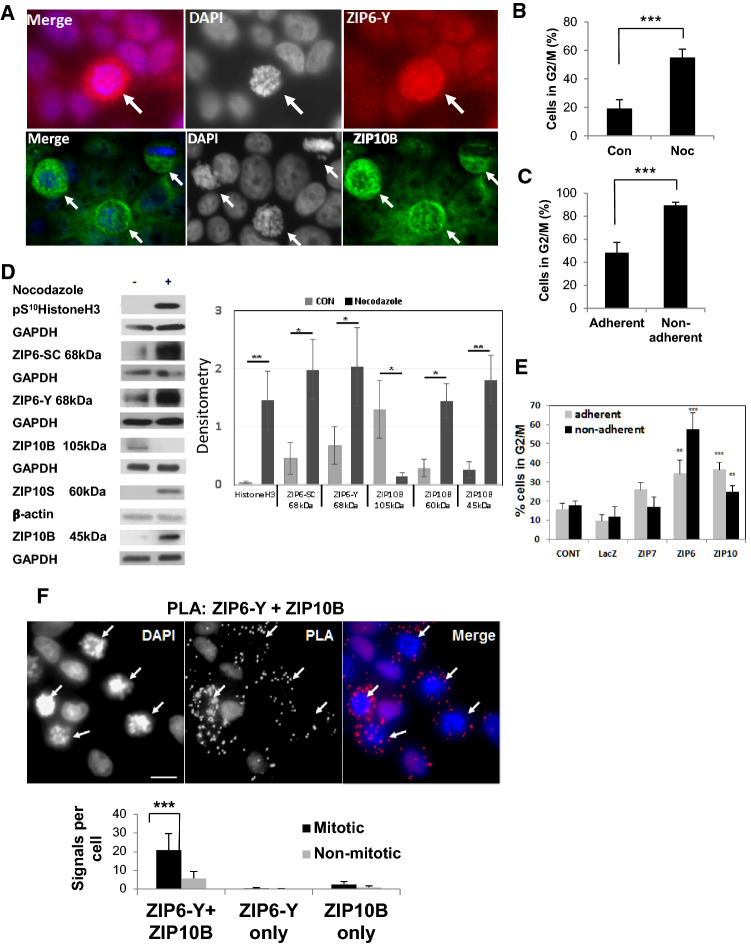
The ZIP6/ZIP10 heteromer is increased in mitosis. **a** Non-permeabilised Mitotic MCF-7 cells (arrow) have increased staining for ZIP6 (red, ZIP6-Y antibody) and ZIP10 (green, ZIP10B antibody) on the plasma membrane in contrast to the non-mitotic cells. Nuclei stained blue with DAPI. **b**, **c** FACS cell cycle analysis confirms the increase compared to control in the G2/M population in nocodazole-treated cells (**b**) as well as the increase in non-adherent cells collected after mitotic shake-off (**c**), compared to the adherent cells from the same dishes. Results of three independent experiments are demonstrated as mean ± SD. Statistical significance is determined by Student’s *t* test.***p < 0.001. **d** MCF-7 cells treated with nocodazole for 20 h have increased mitosis as judged by mitotic marker pS^10^HistoneH3 as shown from a representative blot of more than three experiments. ZIP6 is significantly increased in mitotic cells and the active N-terminal-cleaved form of ZIP6 as a 68 kDa band is recognised by both ZIP6 antibodies (ZIP6-Y recognises N-terminus and ZIP6-SC recognises the cytoplasmic loop between TM3-4). ZIP10 undergoes cleavage in mitosis; Using the N-terminal antibody ZIP10B the full length protein (105 kDa) decreased in the nocodazole-treated population and the smaller cleaved fragment increased significantly (45 kDa). Using the cytoplasmic loop antibody ZIP10S, an increase in the 60 kDa band was observed in mitosis which corresponded to the remainder of ZIP10 after removal of some of the N-terminus. Statistics (student *t* test) were compared to control. **e** FACS cell cycle analysis of transfected cells, divided into adherent and non-adherent populations after mitotic shake off showing that transfection with ZIP6 or ZIP10 increased the cells in G2/M whether from the adherent on nonadherent populations. Results of at least three independent experiments are shown as mean ± SD Statistical significance comparing results to control samples was performed using ANOVA with Dunnett post-hoc and is shown as ***(*p* < 0.01) or ***(*p* < 0.001). **f** Proximity ligation assay (PLA) using ZIP6-Y and ZIP10B antibodies in nocodazole-treated MCF-7 cells, produces significantly increased dots (red) in mitotic cells (white arrows) compared to non-mitotic cells. Nuclei stained with DAPI (blue). Representative picture of quantitative measurements from at least 6 images of 25 stacks taken 0.3 μm apart from 3 independent experiments are demonstrated as mean ± standard error. Statistical significance was compared between mitotic and non − mitotic cells using ANOVA with Dunnett post-hoc and is shown as ***p < 0.001. Scale bar, 25 μm

The ZIP6 protein is not only highly regulated in cells but also responsible for causing cell rounding [23], an essential early component of mitosis. Using antibodies with epitopes on the extracellular N-terminus, both ZIP6 (red) and ZIP10 (green) are visible preferentially on the outside of non-permeabilised mitotic cells (Fig. 1a, white arrows) while generally absent from non-mitotic cells. Interestingly, all the ZIP6 and ZIP10 positive cells are in the prophase stage of mitosis with the exception of one of the ZIP10 positive cells (Fig. 1a, top right) which is in metaphase, consistent with the presence of the relevant N-terminal sections at this stage of mitosis. The number of mitotic cells is enhanced by 20 h of nocodazole treatment, an agent that blocks microtubule polymerisation, as judged by FACS cell cycle analysis, which demonstrates an increased number of cells in G2/M in the whole population (Fig. 1b) and in the non-adherent cells after mitotic shake off (Fig. 1c). Using these conditions we saw significantly increased levels of both ZIP6 and ZIP10 in mitosis, as judged by increased pS^10^HistoneH3 (Fig. 1d) in nocodazole treated samples compared to untreated control conditions. The 68 kDa band represents the N-terminally cleaved and active form of ZIP6 located on the plasma membrane [23], as recognised by the N-terminal directed antibody, ZIP6-Y (with epitope downstream of this cleavage site), and also the ZIP6-SC antibody, which recognises the cytoplasmic loop between TM3-4 [23]. ZIP10 undergoes N-terminal ectodomain shedding in the presence of nocodazole, represented by a decrease in the full-length protein and an increase in a 45 kDa fragment, corresponding to a large portion of the N-terminus, as recognised by the N-terminal ZIP10B antibody. The epitope of the ZIP10S antibody recognises the cytoplasmic loop between TM3-4 and therefore was able to recognise an increase in the 60 kDa band which represents the full length ZIP10 after part of the N-terminus has been cleaved. Furthermore, as we had previously discovered that transfecting cells with ZIP6 or ZIP10 increased the population of mitotic cells twofold [29], we expanded this to incorporate a mitotic shake off, enabling enrichment of the non-adherent, loosely attached population of mitotic cells. Examining the adherent cell populations, we demonstrate an increase in mitotic cell number in cells transfected with ZIP6 or ZIP10 (Fig. 1e) compared to LacZ control or ZIP7, used as a control ZIP protein. This increase in mitotic cell number was also seen in non-adherent cells after transfection with ZIP6 or ZIP10 (Fig. 1e) compared to controls, with the non-adherent ZIP6 transfected cells increasing their mitotic cell number fourfold. This data confirms the role of ZIP6 and ZIP10 in initiating mitosis.

We next examined the binding of ZIP6 and ZIP10 in mitotic cells using proximity ligation assay, a quantitative method that generates red fluorescent dots if two molecules are close enough to be likely to interact [13]. Using this method, we show that ZIP6 and ZIP10 heteromers exist preferentially in mitotic cells (Fig. 1f, white arrows) compared to non-mitotic cells and controls (supplementary Fig. A).

**Fig. 2:**
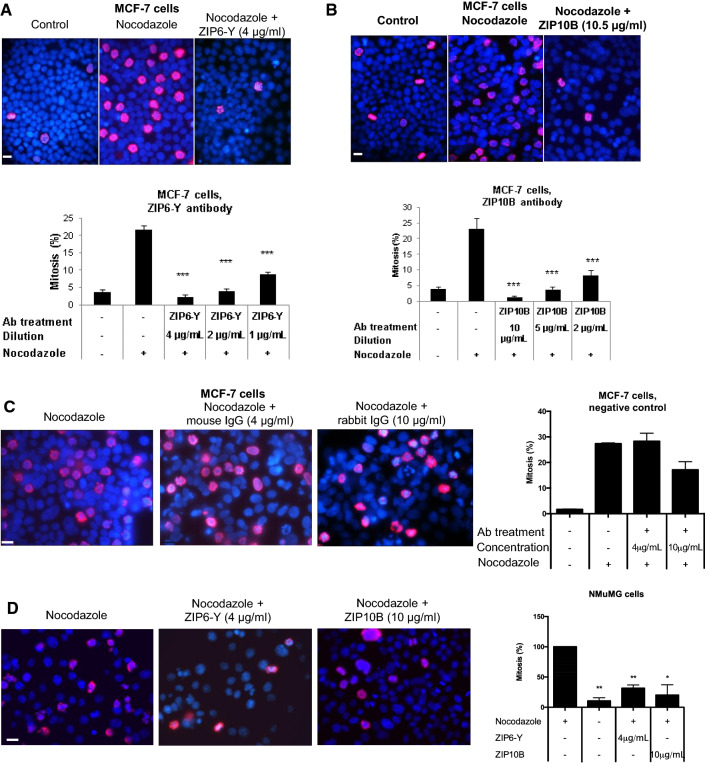
The ZIP6/ZIP10 heteromer is essential for mitosis. Treatment of MCF-7 cells with 100 nM nocodazole for 20 h and either ZIP6-Y (**a**) or ZIP10B (**b**) antibodies significantly decreases the number of mitotic cells (positive for pS^10^HistoneH3, red) in a concentration-dependent manner which was not observed with treatment with the relevant IgG controls (**c**) for mouse ZIP6-Y and rabbit ZIP10B antibodies. Treatment of NMuMG cells with 100 nM nocodazole for 20 h with either ZIP6-Y or ZIP10B antibodies significantly decreases the number of mitotic cells (**d**). All experiments were repeated at least three times. Statistical significance was compared to nocodazole-treated cells, using ANOVA with Dunnett post-hoc and is shown as *p < 0.05, **p < 0.01, ***p < 0.001

**Fig. 3:**
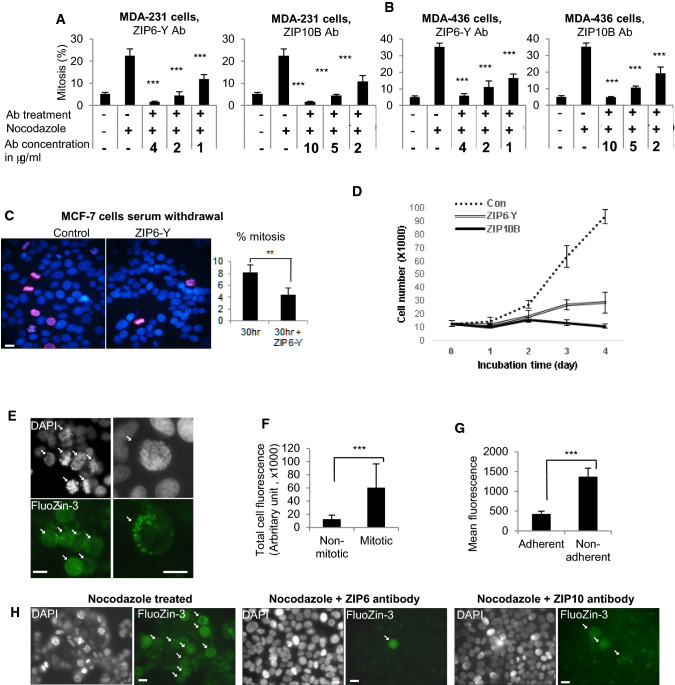
The ZIP6/ZIP10 heteromer is essential for mitosis in different cell lines. Treatment of MDA-231 (**a**) or MDA-436 (**b**) cells with ZIP6-Y or ZIP10B antibody with nocodazole for 20 h significantly decreases the number of mitotic cells (positive for pS^10^HistoneH3, red). **c**. MCF-7 cells, synchronised by 24-h serum withdrawal, were tested 30 h after serum replacement, the time it takes to enter mitosis after synchronisation. Mitotic count, positivity for pS^10^HistoneH3 (red), reveals a significant decrease in the number of mitotic cells due to ZIP6-Y antibody treatment ( 4 μg/ml). **d** Cell growth is significantly suppressed by treatment with either ZIP6-Y (4 μg/ml) or ZIP10B (10.5 μg/ml) antibody over 96 h. ** e** MCF-7 cells loaded with 5 μM Fluozin-3 have increased green fluorescence only in mitotic cells (white arrows), as judged by DAPI staining (greyscale). **f** A significant increase of green fluorescence in mitotic cells was confirmed by FACS analysis of total cell fluorescence of cells stained with Fluozin-3. Mitotic cells were separated using pS^10^HistoneH3. **g** FACS analysis of nocodazole-treated MCF-7 cells loaded with Fluozin-3, show increased green fluorescence in the non-adherent population that had been removed by mitotic shake-off. **h** MCF-7 cells were treated with nocodazole for 20 h either alone or in conjunction with the ZIP6Y antibody at 4 μg/ml or the ZIP10B antibody at 10 μg/ml. Cells were loaded with 5 μM Fluozin-3 and increased green fluorescence was only seen in mitotic cells (white arrows), as judged by DAPI staining (greyscale). The antibody treated cells had much less mitotic cells than nocodazole alone treated cells. All experiments are **r**esults of at least three independent experiments and are demonstrated as mean ± SD. For **a**–**c**, statistical significance is compared to nocodazole-treated samples using ANOVA with Dunnett post-hoc.**p < 0.01, ***p < 0.001. Scale bar, 10 μm

We then hypothesised that our ZIP6-Y or ZIP10B antibodies, recognising the extracellular N-termini of ZIP6 or ZIP10, respectively, are able to block the zinc transport across the ZIP6/ZIP10 heteromer and thus prevent mitosis initiation. In order to test this, we first treated cells with nocodazole to demonstrate the increased percentage of mitotic cells, as judged by the number of pS^10^HistoneH3 positive cells (Fig. 2, red). Subsequent treatment of cells with either our ZIP6-Y (Fig. 2a) or ZIP10B (Fig. 2b) antibody (both N-terminal epitopes) in addition to nocodazole significantly reduced the number of cells in mitosis in an antibody concentration-dependant manner (Fig. 2a, b, graphs), suggesting the ability of our antibodies to indeed block ZIP6/ZIP10 heteromer mediated zinc influx. Furthermore, addition of the relevant IgG at 4 μg/ml (mouse as control for ZIP6-Y) or 10.5 μg/ml (rabbit as control for ZIP10B) to nocodazole treated cells (Fig. 2c) had no effect on the mitotic index of the cells, confirming a ZIP6/ZIP10-dependant effect. The role of ZIP6 and ZIP10 in mitosis is also relevant to non-cancerous cells, as treatment with both the ZIP6-Y and ZIP10B antibodies inhibits mitosis in NMuMG (normal mouse mammary gland cells) (Fig. 2d). This result was also reproduced in two triple negative (ER-, PR- and Her2-) breast cancer cell lines, MDA-231 (Fig. 3a) and MDA-436 (Fig. 3b).

To confirm that blocking ZIP6 did not work by stabilising the microtubules and thus helping the cells to overcome the nocodazole block, we repeated this experiment in the absence of nocodazole. In order to achieve this, we partially synchronised the cells by serum withdrawal for 24 h and observed that cells entered mitosis 30 h later. Addition of the ZIP6-Y antibody to these synchronized cells significantly decreased mitosis (Fig. 3c) in the absence of nocodazole. Additional confirmation that nocodazole was not involved was obtained by cell treatment with ZIP6-Y or ZIP10B antibody for 4 days (Fig. 3d) demonstrating the ability of the ZIP6/ZIP10 heteromer blocking antibodies to significantly suppress cell growth and confirming the requirement of ZIP6/ZIP10-mediated zinc influx to trigger mitosis.

We next confirmed an increase of intracellular zinc ions in mitotic cells (Fig. 3e), by imaging zinc using the zinc-sensitive fluorescent agent Fluozin-3. White arrows indicate mitotic cells which all had increased fluorescence consistent with an increase in available free Zn^2+^ ions. A significant increase in [Zn^2+^]_i_ in mitotic cells compared to non-mitotic cells was confirmed by FACS analysis, separating the mitotic cells using pS^10^HistoneH3 (Fig. 3f). Furthermore, after mitotic shake off, we observed a threefold increase in Fluozin-3 fluorescence in non-adherent cells (Fig. 3g) which were enriched with mitotic cells, as 90% had 4 N DNA content (Fig. 1c) compared to 48% for adherent cells. We also show an increase of [Zn^2+^]_i_ in the mitotic cells of MCF-7 cells that had been treated with nocodazole for 20 h (Fig. 3h), by imaging zinc using the zinc-sensitive fluorescent agent Fluozin-3. Similarly, cells treated with nocodazole and additionally either ZIP6 or ZIP10 antibody also showed increased green fluorescence in mitotic cells, although the overall number of mitotic cells present in the antibody treated samples was much less, consistent with the results shown in Fig. 2 and suggestive that cells require zinc influx before they can progress to mitosis.

### The ZIP6/ZIP10 heteromer binds phosphorylated STAT3 (pS^727^STAT3) in mitosis

**Fig. 4:**
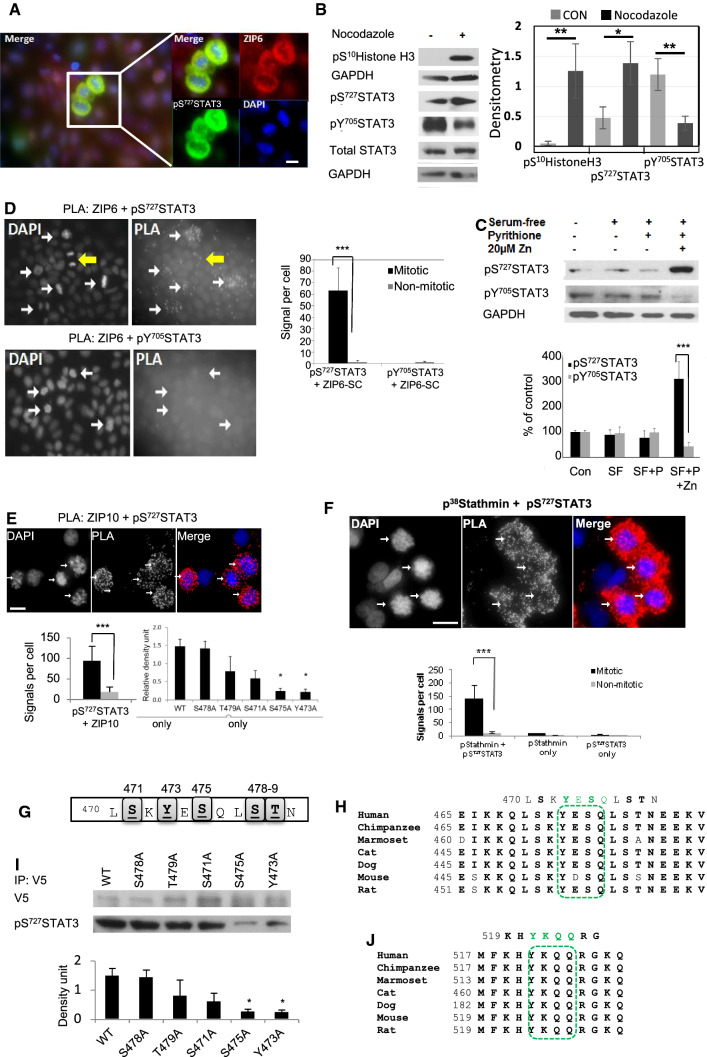
Both ZIP6 and ZIP10 bind pS^727^STAT3 in mitosis. **a** Mitotic MCF-7 cells, positive for ZIP6-SC (red), pS^727^STAT3 (green) and DAPI (blue), and above the plain of adherent cells, are enriched for ZIP6 and pS^727^STAT3 (inset). **b** There was a Significant increase in pS^727^STAT3 and decrease in pY^705^STAT3 when mitosis was increased by nocodazole treatment (student t-test statistics). **c** A significant zinc-dependent increase in pS^727^STAT3 in MCF-7 cells treated with 20 μM zinc (Zn) and 10 μM pyrithione (P) in serum-free medium (SF) for 20 min and a corresponding decrease in pY^705^STAT3. **d** PLA using ZIP6-SC and pS^727^STAT3 antibodies in nocodazole-treated MCF-7 cells produces a significantly increased number of dots only in mitotic cells (white arrows), in contrast to ZIP6-SC and pY^705^STAT3 antibodies which show negligible dots. Yellow arrow indicates less dots in cytokinesis. **e** PLA using pS^727^STAT3 and ZIP10 cytoplasmic loop antibodies in nocodazole-treated MCF-7 cells produces a significant increase in dots in mitotic cells (white arrows). **f** PLA using pS^38^Stathmin and pS^727^STAT3 antibodies in nocodazole-treated MCF-7 cells produces increased dots only in mitotic cells (white arrows). **g** Schematic of residues around the STAT3-binding site in ZIP6 (Y473) showing potential phosphorylation sites. **h** The predicted STAT3-binding site YESQ, residues 473–476, in ZIP6 (green box) is conserved in different species. **i** Immunoprecipitation with V5 antibody in cells transfected with ZIP6 wild-type (WT) or sequence-verified mutants (Fig. S2A-B), all with a C-terminal V5 tag, demonstrates a significant decrease in binding to pS^727^STAT3 in S475A and Y473A mutants. **j** Predicted STAT3-binding site YKQQ, residues 521–524, in ZIP10 (green box) is conserved in different species. All results in this figure represent at least three independent experiments and graphs are mean ± SD. Statistical significance was performed using ANOVA with Dunnett post-hoc and is shown as *(*p* < 0.05), **(*p* < 0.01) or ***(*p* < 0.001). Scale bar = 10 μm

We observed that when ZIP6 was enriched in rounded mitotic cells (Fig. 4a), these cells also contained pS^727^STAT3 (Fig. 4a). We confirmed a significantly elevated pS^727^STAT3 in mitosis (Fig. 4b), by Western blot with a concurrent reduction of pY^705^STAT3 when cells had been treated with nocodazole. Furthermore, we confirmed that zinc treatment for 20 min reduces the amount of pY^705^STAT3, as observed by others [40], and additionally increases the amount of pS^727^STAT3 (Fig. 4c), demonstrating a reciprocal relationship between pY^705^STAT3 and pS^727^STAT3.

In order to identify the relevance of pS^727^STAT3 to the mechanism of mitosis initiation, we next investigated whether this form of STAT3 was bound to ZIP6 or ZIP10 during mitosis using proximity ligation assay. We were able to demonstrate significant binding of pS^727^STAT3 to both ZIP6 (Fig. 4d) and ZIP10 (Fig. 4e) exclusively in mitotic cells with no evidence of pY^705^STAT3 presence in mitotic cells (Fig. 4d and S3A). In contrast there was no detectable interaction between ZIP6 and pY^705^STAT3 in any cells (Fig. 4d, S1D). Interestingly, there was a reduction of pS^727^STAT3 bound to ZIP6 after cytokinesis (Fig. 4d, yellow arrow) suggesting dissociation of STAT3 from the complex after mitosis (Supplementary Figure S3A). These data together establish that the ZIP6/ZIP10-mediated zinc influx at the start of mitosis changes the phosphorylation state of STAT3 from pY^705^STAT3 to pS^727^STAT3 which then binds in a complex to ZIP6 and ZIP10 exclusively during mitosis.

Interestingly, STAT3 is already known to bind to p^38^Stathmin [41]. This form of Stathmin is present in mitosis not only to enable the microtubule reorganisation that is required for mitosis [41] but also as an essential requirement for both mitotic spindle assembly [42] and mitotic entry [39]. Having demonstrated that pS^727^STAT3 and pS^38^Stathmin co-localise throughout the different stages of mitosis (Figure S4A) we also confirmed, by proximity ligation assay, that pS^727^STAT3 and pS^38^Stathmin bind exclusively in mitotic cells (Fig. 4f, S4B), supporting a role for pS^727^STAT3 in stabilising Stathmin throughout mitosis and integrating the ZIP6/ZIP10/pS^727^STAT3/ pS^38^Stathmin complex into the established mitotic cascade.Using the ELM server [43] we discovered that the ZIP6 protein sequence has a predicted STAT3-binding site on the cytoplasmic loop (YESQ, residues 473–476) between transmembrane domains 3–4, which fits the consensus motif of a STAT3-binding site YxxQ [44] (Fig. 4g) and is highly conserved in the ZIP6 sequence in mammals (Fig. 4h). We generated a ZIP6 mutant Y473A to abolish this binding site and tested this by immunoprecipitation (Fig. 4i, S2A, S2B). Probing for pS^727^STAT3 in cells immunoprecipitated with V5 in cells transfected with both wild-type ZIP6 and other ZIP6 mutants showed reduced presence in the Y473A mutant (Fig. 4i), consistent with STAT3 binding to ZIP6 involving residue Y473. Interestingly the nearby S475A mutant also showed an effect suggesting a potential role for phosphorylation in the binding. ZIP10 also has a well conserved predicted STAT3-binding site YKQQ (512–524) in the corresponding region (Fig. 4j) suggesting the potential for ZIP10 binding as well. These data demonstrate the presence of pS^727^STAT3 in mitotic cells which binds to both ZIP6 and ZIP10.

**Fig. 5:**
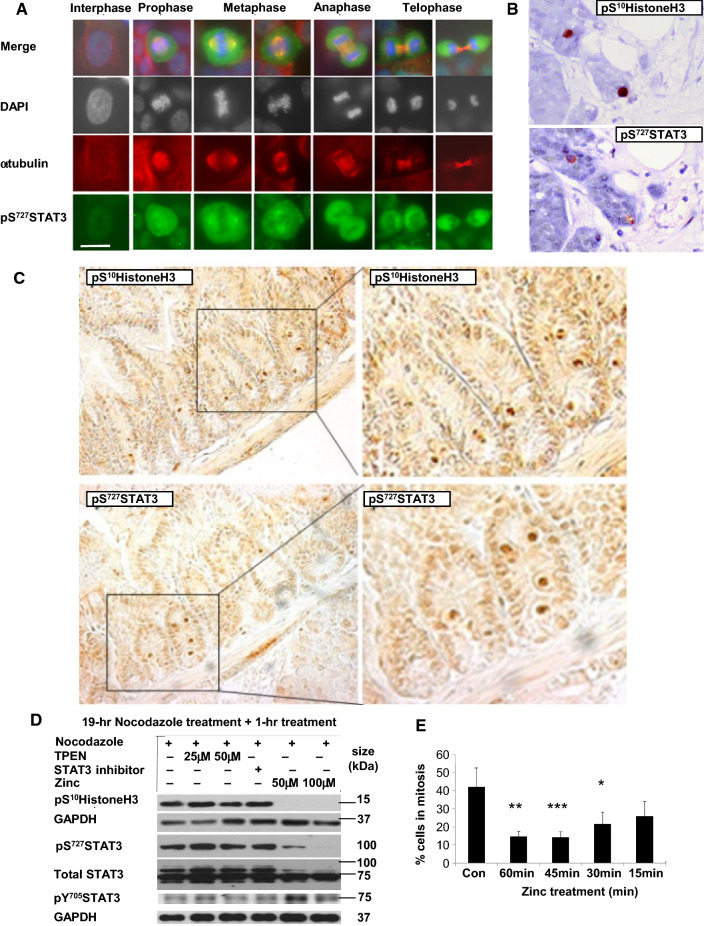
pS^727^STAT3 is present throughout mitosis and is cleaved for mitosis exit. **a** pS^727^STAT3 (green) is increased in all stages of mitosis in cells stained with DAPI (blue) and α-tubulin (red) compared to interphase. Scale bar = 10 μm. **b** Adjacent slices of breast cancer tissue show both pS^10^HistoneH3 and pS^727^STAT3 in the same mitotic cells (red staining). **c** Both pS^10^HistoneH3 and pS^727^STAT3 are present in mitotic cells within the crypts of normal mouse intestine (brown staining). **d** Reduction of zinc (zinc chelator TPEN) or STAT3 inhibition for 1 h after cells were pre-treated with nocodazole for 19 h has no effect on mitosis whereas zinc–treated samples move through mitosis faster, loosing pS^10^HistoneH3 and pS^727^STAT3. Full-length STAT3 is C-terminally cleaved when cells move out of mitosis (zinc-treated lanes), physically removing residue S727. **e** Mitotic count (positive for pS^10^HistoneH3) was performed in cells treated with nocodazole for 19 h plus 100 μM zinc and 10 μM pyrithione for 0-60mins. Cells begin to progress through mitosis after only 15 min zinc treatment**.** Results are from at least three experiments. Statistical significance comparing the zinc treatment to the non- treated samples (CON) was performed using ANOVA with Dunnett post-hoc and is shown as *(*p* < 0.05) or **(*p* < 0.01)

To dissect the temporal association of pS^727^STAT3 with the mitotic process, we imaged pS^727^STAT3 in cells during mitosis (Fig. 5a). This revealed the presence of pS^727^STAT3 at all stages of mitosis which was evident before cells were positive for pS^10^HistoneH3 and also in cytokinesis when cells were still positive for pS^727^STAT3 yet negative for pS^10^HistoneH3, indicating the prolonged presence of pS^727^STAT3 throughout mitosis compared to pS^10^HistoneH3 (Figure S3A, middle panel, white arrow). In contrast, pY^705^STAT3 was absent in all mitotic cells (Figure S3A).

Furthermore, we also detected pS^727^STAT3 staining in mitotic cells in vivo*,* as judged by pS^10^HistoneH3 in the same cells using adjacent slices of human breast cancer (Fig. 5b). Additional in vivo staining of pS^727^STAT3 was also seen in the mitotic cells of normal mouse intestine (Fig. 5c), suggesting a common process during mitosis that encompasses normal and disease states.

We have also gained some novel insight into how the S727 phosphorylation of STAT3, required during mitosis, is eliminated efficiently at the end of mitosis by removal of the C-terminus of STAT3, containing residue S727, by proteolytic cleavage. We examined the effect of zinc on mitotic progression by treatment with either a zinc chelator or zinc and also treatment with a STAT3 inhibitor for the last hour of nocodazole treatment. The treatment with zinc chelator or STAT3 inhibitor after treatment with nocodazole had no effect on pS^10^HistoneH3 or STAT3 phosphorylation status (Fig. 5d and S3B for densitometry) once the cells had achieved mitosis, establishing that both STAT3 and zinc were required before cells reached mitosis. However, 1-h incubation with 50 μM or 100 μM zinc after nocodazole treatment had effects consistent with cells no longer in mitosis, as judged by loss of pS^10^HistoneH3 and also the reversion of pS^727^STAT3 to pY^705^STAT3 (Fig. 5d). We then confirmed that these zinc-treated cells had progressed quicker through mitosis using a reduced zinc exposure time (Fig. 5e), noticing a loss of mitotic cell number as soon as 15 min after zinc treatment. Importantly, these zinc-treated cells which had exited mitosis had lost the usual full length STAT3 band (Fig. 5d), showing only the C-terminally cleaved form of STAT3, thus removing a peptide containing residue S727 which is near the C-terminus [45]. This data demonstrates how C-terminal cleavage of STAT3 at the end of mitosis can efficiently stop S727 phosphorylation of STAT3 by removal of this residue and allow residue Y705 to be available for phosphorylation, enabling the active transcription factor form of STAT3 to be re-instated.

### Cells require both ZIP6 and ZIP10 for maximal growth rate

**Fig. 6:**
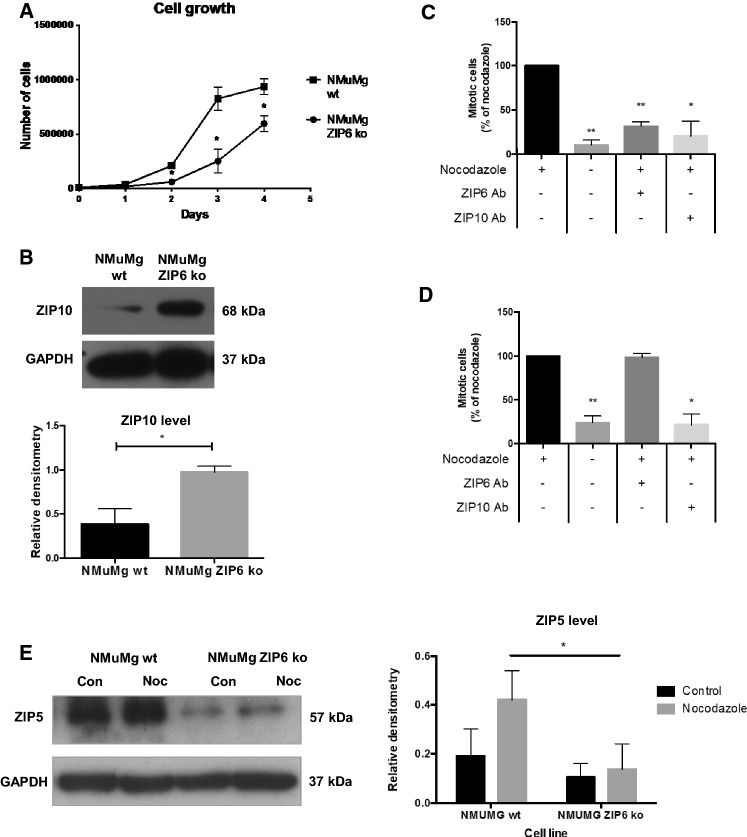
Cells require both ZIP6 and ZIP10 for maximal growth rate. **a** ZIP6 knockout NMuMg cells (NMuMg ZIP6 ko) grow significantly slower than wild-type cells. **b** ZIP10 level is significantly increased in NMuMg ZIP6 knockout cells compared to wild-type by Western Blot. Both ZIP6 and ZIP10 antibodies are able to significantly inhibit mitosis in NMuMg wild-type cells (**c**) whereas ZIP6 antibody is unable to inhibit mitosis in ZIP6 knockout cells and ZIP10 antibody can (**d**).** e** Western blot comparing levels of ZIP5 in wild-type and ZIP6 knockout NMuMg cells. There is a significant reduction in ZIP5 levels in the ZIP6 knockout cells even in the presence of nocadazole. All results are from at least three experiments. Statistical significance comparing the results to controls was performed using ANOVA with Dunnett post-hoc and is shown as *(*p* < 0.05) or **(*p* < 0.01)

In order to further examine the relationship between ZIP6 and ZIP10 in the process of mitosis initiation, we compared NMuMg mouse breast cells to those that had ZIP6 removed by crispr/Cas9 technology [46]. Specifically, we show that NMuMg cells with ZIP6 knockout grow slower than wild-type cells (Fig. 6a) and have considerably increased ZIP10 levels compared to the wild-type cells (Fig. 6b), suggesting that the cells are able to some degree to compensate for the loss of ZIP6 by upregulating ZIP10. Additional confirmation of this mechanism is provided by the fact that it was possible to inhibit mitosis in wild-type NMuMg cells with either ZIP6 or ZIP10 antibody (Fig. 6c) whereas ZIP6 antibody failed to prevent mitosis in the ZIP6 knockout cells while the ZIP10 antibody treatment inhibited mitosis as expected (Fig. 6d). This adds further weight to the argument that ZIP10 can compensate for loss of ZIP6 to initiate mitosis, allowing the cells to divide, albeit at a lower rate than in the presence of ZIP6.

In order to examine whether any other ZIP family members could be involved in this mitosis mechanism, especially when ZIP6 has been removed, we investigated whether ZIP5 was involved as both ZIP6 and ZIP10 have been previously demonstrated by mass spectrometry screens to bind ZIP5 [47] but no other ZIP transporters. Furthermore, ZIP5 is the only other LIV-1 family member present on the same arm of the SLC39A family phylogenetic tree as ZIP6 and ZIP10 [27] and therefore the prime candidate. We analysed the levels of ZIP5 in these wild-type NMuMg mouse cells to examine whether ZIP5 increased when the cells were treated with nocodazole suggesting that ZIP5 may play a role during mitosis when either ZIP6 and ZIP10 are not available. We discovered that there was no difference between ZIP5 levels in mitotic or non-mitotic conditions. However, analysis of the ZIP5 levels in the ZIP6 knockout cells showed a significant decrease of ZIP5 levels compared to wild-type cells (Fig. 6e) suggesting that ZIP5 is not involved in the growth of these cells and therefore unlikely to be partnering with ZIP10 to drive mitosis.

### Inhibition of ZIP6/ZIP10 heteromer-induced mitosis leaves cells in G2

**Fig. 7:**
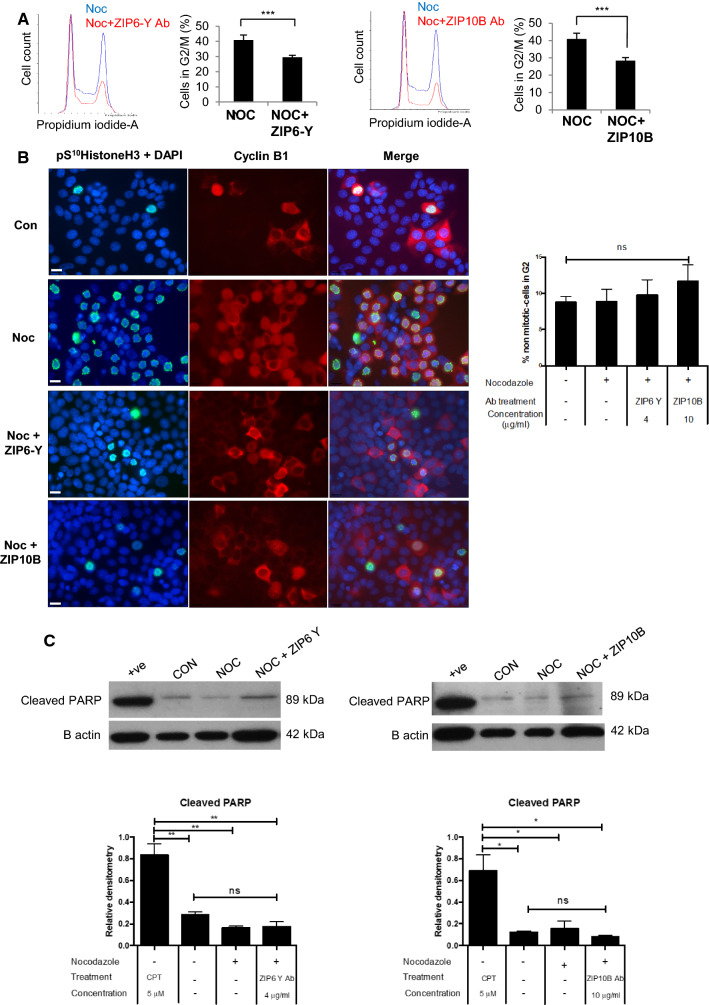
ZIP6 or ZIP10 inhibited cells remain in G2 and are not apoptotic. **a** FACS cell cycle analysis reveals a significant decrease in the G2/M population in MCF-7 cells treated with either ZIP6-Y (2 μg/ml) or ZIP10B (5.25 μg/ml) antibody. The graphs show the mean of *n* = 3 ± SE. **b** Imaging differences between mitotic cells (pS^10^HistoneH3, green) and those in G2/M stages of the cell cycle (cyclinB1, red) when treated with ZIP6-Y antibody, ZIP10B antibody and/or nocodazole for 20 h. The percentage of cells positive for cyclin B1 is shown as a bar graph. No statistical significance was revealed. **c** There is no increase in apoptotic cells when treated with ZIP6-Y (4 μg/ml) or ZIP10B (10.5 μg/ml) antibodies as judged by Western Blot of cleaved PARP and a positive control of apoptosis (camptothecin 5 μM). All results are from at least three experiments. Statistical significance comparing the + ve to the different cell treatment and the non- treated samples (CON) was performed using ANOVA and is shown as *(*p* < 0.05) or **(*p* < 0.01). No statistical significance was found between the samples treated with nocodazole ± ZIP6 or ZIP10 antibody and the non-treated samples (CON)

Demonstrating that ZIP6-Y antibody and ZIP10 antibody treatments could inhibit cell growth we next investigated what happens to the antibody-treated cells that do not enter mitosis. FACS cell cycle analysis revealed a significant decrease in the G2/M population in MCF-7 cells treated with either the ZIP6-Y antibody (1:20 or 4 μg/ml) or the ZIP10B antibody (1:20 or 10 μg/ml) (Fig. 7a). The previously observed decrease in mitosis in this population would suggest an increase of cells in G2. We assessed this by imaging for cyclin B1, a marker of mitosis and G2, in cells that had been treated with these antibodies using single cell fluorescence analysis (Fig. 7b). There was no statistical difference between the groups, neither was there any increase in apoptosis as judged by Western Blot of cleaved PARP on the same gel (Fig. 7c). We conclude from this data that cells treated with nocodazole and the ZIP6 or ZIP10 antibody have not become apoptotic but have remained in the G2 stage of the cell cycle.

All this data together demonstrates a novel mechanism explaining the role of zinc in triggering mitosis. Influx of zinc into cells through the ZIP6/ZIP10 heteromer changes the phosphorylation of STAT3 and sets in motion the assembly of a complex of proteins leading to microtubule reorganisation and chromosome condensation, two key elements required for successful mitosis progression.

## Discussion

**Fig. 8:**
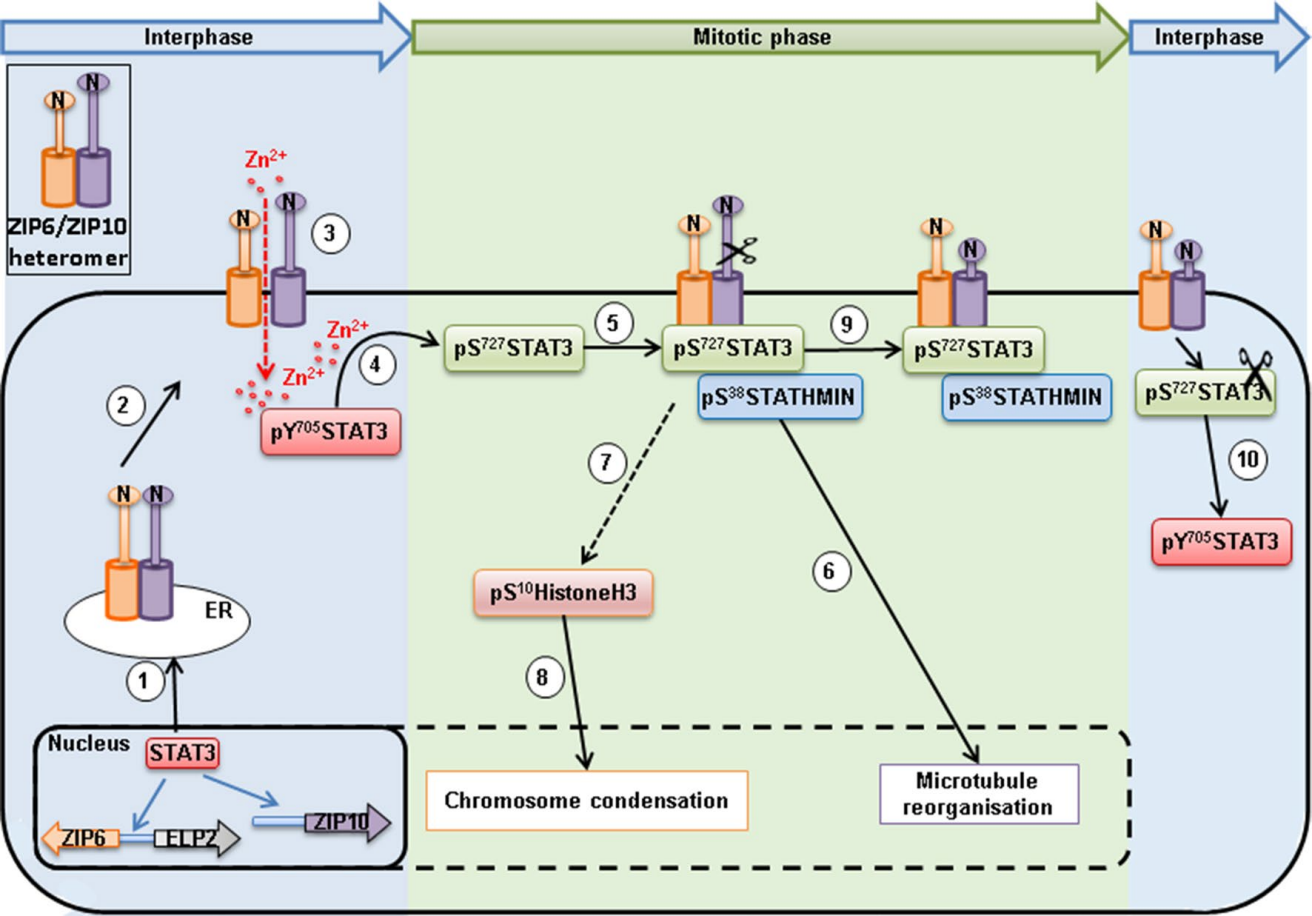
Schematic of how zinc triggers mitosis. Schematic demonstrating a model of how the ZIP6/ZIP10 heteromer imports zinc into cells to trigger mitosis. STAT3 driven ZIP6 [1] is located on the endoplasmic reticulum membrane where it forms a heteromer with ZIP10 [29] until the correct stimulus [2] for ZIP6 N-terminal cleavage and relocation to the plasma membrane [23]. Plasma membrane located ZIP6/ZIP10 heteromer causes cell rounding and influxes zinc to trigger mitosis [3]. This zinc triggers formation of pS^727^STAT3 from pY^705^STAT3 [4], which binds to the ZIP6/ZIP10 heteromer [5]. The binding of pS^38^Stathmin to pS^727^STAT3 as part of the ZIP6/ZIP10 heteromer mitosis complex enables the pS^38^Stathmin-driven microtubule re-organisation needed for mitosis [6] and the activation of pS^10^HistoneH3 [7] which leads to condensation of chromosomes [8]. Between prophase and metaphase, ZIP6 is N-terminally cleaved for a second time and the mitosis complex remains during mitosis [9]. The C-terminus of STAT3 is cleaved at the end of mitosis to remove Ser727 [10] allowing Tyr705 to become phosphorylated and restore STAT3 transcriptional activity

Over fifty years after the original discovery that zinc depletion arrests the cell cycle [48], we propose a molecular role of zinc in mitosis (Fig. 8). There is the formation of a zinc-dependant mitotic complex consisting of ZIP6, ZIP10, pS^727^STAT3 and pS^38^Stathmin that feeds into known mitotic pathways such as Stathmin-dependant microtubule reorganisation and HistoneH3-mediated chromosome condensation. Initially, STAT3 serves as a transcription factor (Fig. 8, stage 1) driving the gene expression of ZIP6 and ZIP10 [49] [23], which forms a heteromer with ZIP10 in the endoplasmic reticulum [29]. The N-terminal cleavage of ZIP6 is required for the ZIP6/ZIP10 heteromer to move to the plasma membrane (Fig. 8, stage 2) to initiate cell rounding [23] and import zinc into cells to start mitosis (Fig. 8, stage 3). This imported zinc triggers the formation of pS^727^STAT3 from pY^705^STAT3 (Fig. 8, stage 4), which remains bound to the ZIP6/ZIP10 heteromer throughout mitosis, preventing STAT3 transcriptional activity which is known to be turned off during mitosis [50]. Thus, STAT3 in addition to being a transcription factor can moonlight as an effector of this heteromer and stabilise pS^38^Stathmin throughout mitosis (Fig. 8, stage 5) which is critical for the formation of normal mitotic spindles [42]. The binding of pS^727^STAT3 and pS^38^Stathmin, confirmed by others [41], with the ZIP6/ZIP10 heteromer in a complex in mitosis, links pS^727^STAT3 to the p^38^Stathmin-driven microtubule re-organisation that is needed during mitosis (Fig. 8, stage 6) and the pS^10^HistoneH3 activation (Fig. 8, stage 7) that leads to chromosome condensation essential for mitosis (Fig. 8, stage 8).

The complex of ZIP6/ZIP10/pS^727^STAT3/pS^38^Stathmin remains until the end of mitosis (Fig. 8, stage 9) when STAT3 is cleaved at the C-terminus, physically removing residue Ser727 (Fig. 8, stage 10), allowing STAT3 to revert to Tyr705 phosphorylation, the active transcription factor form of STAT3. This discovery therefore provides the mechanism of how zinc is the essential trigger for mitosis and the ability of our antibodies to prevent this crucial zinc influx now provides novel new targets for proliferative diseases such as cancer.

Our data confirm previous observations that zinc can bind to STAT3 resulting in decreased pY^705^STAT3 [40] yet we also show a reciprocal increase in pS^727^STAT3. This is the first time that pS^727^STAT3 has been coupled to a mechanism for driving mitosis. Interestingly, the presence of pS^727^STAT3 in mitosis has been observed by a whole genome mass spectrometry screen [51] of protein phosphorylation events during mitosis, consistent with the data presented here. We also detected an inverse association of pY^705^STAT3 and pS^727^STAT3, which has been previously reported by others [52], although mitosis was not examined. Interestingly, transcription ceases during mitosis [53] and loss of pY^705^STAT3, as mediated by zinc in mitosis, will terminate STAT3 transcriptional activities, preventing downstream signalling effects on multiple pathways for the duration of mitosis.

Most focus on STAT3 signalling is centred on pY^705^STAT3, the transcription-promoting form of the protein [54–56], which drives many adverse cancer features such as epithelial-mesenchymal transition in HER2-positive breast tumours [57]. In contrast, pS^727^STAT3 has been widely overlooked [58] despite having an essential role in mouse embryonic and perinatal growth [59] and in proliferation, optimal pluripotency and transition to neural commitment of mouse embryonic stem cells [60]. Recently, however, pS^727^STAT3 was discovered to promote prostate cancer [61] and chronic lymphoid leukaemia [62] independently of pY^705^STAT3 and together with pY^705^STAT3, pS^727^STAT3 associates with poor clinical outcome in glioblastoma [63]. Furthermore, the appearance of pS^727^STAT3 at the start of mitosis suppresses the expression of G1 cell cycle negative regulators p21^CIP1/WAF1^ and p27^Kip1^, maintaining CDK1 activity during mitosis and providing evidence for a role in mitotic onset and progression [64]. Recently, the level of pS^727^STAT3 in glioblastoma has been positively correlated with poor outcome [63], consistent with a role for pS^727^STAT3 in mitosis. Despite these data and the knowledge that most cancers have increased expression of STAT3, no molecular mechanism has previously linked STAT3 to involvement in the onset of mitosis.

The discovery of the role of the ZIP6/ZIP10 heteromer in triggering mitosis has enabled us to use ZIP6 or ZIP10 blocking antibodies to prevent mitosis, an important discovery to help target cell division in diseases of aberrant proliferation, including cancer. These antibodies bind to the extracellular portion of the ZIP6/ZIP10 heteromer on the plasma membrane and each of them alone was able to block the mitosis-triggering zinc influx in a concentration-dependent manner confirming the vital nature of this zinc influx to mitosis initiation. Importantly, the IgG isotype control did not have any effect on mitosis, confirming a ZIP6/ZIP10 specific effect. The new crystal structure of a ZIP transporter [65], has produced a predicted structure for the extracellular region of the ZIP4 dimer [66] which confirmed the essential nature of this extracellular region for full zinc transport activity and explains how the ZIP6 or ZIP10 antibodies can potentially inhibit the ZIP6/ZIP10 heteromer by binding the N-terminus and either blocking the zinc influx or interfering with activation such as protease cleavage. Since prion proteins have descended from ZIP6 and ZIP10 [67] and as a result show much similarity to the N-terminus of ZIP6 and ZIP10, including the ability to influx zinc into cells [68], it is likely that protease cleavage plays a role in the function of ZIP6 and ZIP10. Interestingly, ZIP6 and ZIP10 antibodies to different epitopes have also been demonstrated to inhibit meiosis [69] showing a mutual need for zinc influx in both cell division processes. We have additionally established that ZIP6/ZIP10 antibody treatment inhibits mitosis in triple negative breast cancer cell lines (Fig. 2), a form of breast cancer with poor treatment and prognosis, offering potential for a new treatment.

Both ZIP6 and ZIP10 independently have been associated with cancers, especially those with poor prognosis, which is consistent with this newly discovered role for ZIP6 and ZIP10 in driving mitosis. ZIP6 has been implicated in oesophageal cancer [70, 71], breast cancers [23] prostate cancer [72], consistently being associated with more aggressive forms of the diseases. Similarly, ZIP10 over-expression has been correlated to aggressiveness in renal cell carcinoma [36], metastatic breast cancer [35], gastric cancer [73] and it is one of the 7 zinc transporter genes upregulated in activated colon tumour cells [24], compatible with our proposed role for ZIP10 in cell division.

Our newly discovered mechanism requires zinc influx specifically through the ZIP6/ZIP10 heteromer into cells to trigger mitosis. We have demonstrated increased fluorescence of the zinc-dependant Fluozin-3 dye in mitotic cells consistent with observations by others using synchrotron x-ray fluorescence microscopy [74] demonstrating a threefold zinc increase in mitotic versus interphase cells. Furthermore, zinc accumulates in the tail tip of zebrafish embryos, the key area contributing to embryonic growth [75], a process that requires much cell division and a further example of the zinc requirement for cell division.

Stathmin is prometastatic in colon cancer, a process that requires pS^38^Stathmin and knockdown of Stathmin was shown to reduce STAT3 [76], confirming an association of these two proteins. Furthermore, as pS^38^Stathmin is critical for the formation of the normal mitotic spindle [42], its binding to pS^727^STAT3 during mitosis will stabilise the form of Stathmin that enables microtubule reorganisation [41], an essential component of the mitosis process. Dephosphorylation of pS^38^Stathmin is required before cells can exit mitosis [42] which suggests a role for STAT3 C-terminal cleavage in dissociating the complex and allowing mitosis exit. C-terminal cleavage of STAT3 will remove residue S727 [45] enabling STAT3 phosphorylation on Y705, the active transcription factor form of STAT3, which is required on exit from mitosis. Furthermore, PP2A, which dephosphorylates pS^727^STAT3 [77] and is inhibited by zinc [78], becomes activated at the end of mitosis when zinc levels decrease [79], providing a secondary mechanism for pS^727^STAT3 removal at the end of mitosis.

Knowledge that STAT3 is required for the expression of both ZIP6 [38] and ZIP10 [29] in the gastrula organiser coupled with our discovery of the involvement of pS^727^STAT3 in mitosis defines the previously unresolved role for this form of STAT3 in cells and explains why STAT3 is the only STAT gene to be embryonic lethal [80]. Importantly, not only do these STAT3 knockout cells die before gastrulation, the stage of embryogenesis that requires ZIP6 [38], STAT3 is expressed in the visceral endoderm, the part of the embryo that takes part in exchange of nutrients such as zinc. Our data combines with this to support the crucial role for ZIP6 as the means to bring zinc into cells to initiate mitosis in a STAT3-dependant manner. Given that STAT3 is known to be phosphorylated on residue S727 by many different kinases [52, 81, 82], it is yet to be determined whether zinc causes pS727 phosphorylation of STAT3 directly or through activation of one of these kinases. We have however established that pS^727^STAT3 binds ZIP6 on residue Y473, a consensus motif of a STAT3-binding site YxxQ [44].

The 3 key molecules in this zinc-driven mitosis pathway, ZIP6, ZIP10 and pS^727^STAT3, now offer new therapeutic opportunities for inhibition of cell division in proliferative diseases such as cancer.

## Materials and methods

### Materials, antibodies and treatments

Antibodies used were ZIP6-SC (E-20, SC-84875), pS^727^STAT3 (SC-8001-R and SC-136193), pY^705^STAT3 (SC-7993-R), total STAT3 (SC-8019), normal mouse IgG (sc-2025), normal rabbit IgG (sc-2027) and GAPDH (SC-32233) from Santa Cruz Biotechnology; α-tubulin (DM1A, #3873S), pS^10^HistoneH3 (#9706S and #3377) and pS^38^Stathmin (#4191) from Cell Signalling Technology; mouse V5 from Invitrogen; rabbit V5 (Ab9116), mouse/rabbit apoptosis cocktail (ab136812) from Abcam; β-actin (A5316), ZIP10 cytoplasmic loop antibody (SAB1401780) from Sigma-Aldrich (referred to as ZIP10S antibody). Treatments used were 100 ng/mL nocodazole (Sigma-Aldrich, M1404) for 20 h, 200 μM STAT3 inhibitor cell-permeable peptide (Calbiochem, 573,096), 5 μM camptothecin (Sigma- Aldrich) for 20 h and 20–100 μM zinc with 10 μM sodium pyrithione (Sigma-Aldrich), and 25 or 50 μM TPEN (Sigma-Aldrich). Mitosis inhibition experiments were performed using mouse monoclonal antibody generated by Biogenes to ZIP6 residues 240–253(ZIP6-Y) and rabbit polyclonal antibody to ZIP10 residues 46–59 (ZIP10B) which were added to cells in culture for 20 h in the presence of nocodazole.

### Cell lines and Immunohistochemistry

MCF-7 cells were cultured as previously described [26]. NMuMG cells were cultured in Dulbecco’s Modified Eagle’s Medium (DMEM) (Gibco) containing 4.5 g/L glucose supplemented with 10% foetal calf serum (FCS), 200 mM l-glutamine, antibiotics (10 IU/mL penicillin, 10 $\mu$g/mL streptomycin) and amphotericin B (2.5 $\mu$g/mL) (Gibco). Formalin-fixed paraffin-embedded breast cancer samples were dewaxed and rehydrated before incubated with pS^727^STAT3 (SC-8001-R, 1/650) or pS^10^HistoneH3 (#3377, 1/30) antibodies for 2 h and detected with Dako Envision #K4011 reagent. Two-minute pressure cooker at pH9 in Tris base plus EDTA was used for antigen retrieval. The primary breast cancer material used had correct ethical approval (REC reference number C2020313). Immunohistochemistry of mouse intestinal tissue was previously described [83].The ZIP6 knockout cells were made from CRISPR-Cas9-edited mouse mammary NMuMG cells as previously described [46] and were a kind gift from G. Schmitt-Ulms.

### Plasmids and transfections

The generation of recombinant constructs for ZIP6/LIV-1/SLC39A6 [84], ZIP7/HKE4 [85] and ZIP10 [29] with C-terminal V5 tags using vector pcDNA3.1/V5-His-TOPO has been previously described. ZIP6 mutants (Y473A, S471A, S475A and S478A, S479A) were generated from the above plasmid and confirmed by sequencing (Figure S2A, S2B). Cells were transfected with Lipofectamine-2000 (Life Technologies) for 16 h as described [23].

### SDS-PAGE, western blotting and immunoprecipitation

Cells were harvested, washed with PBS, lysed for 1 h at 4 °C with lysis buffer pH7.6 (50 mM Tris, 150 mM NaCl, 5 mM EGTA and 1% Triton X-100) with protease inhibitor cocktail for mammalian cells (Sigma-Aldrich) and phosphatase inhibitors (2 mM sodium orthovanadate and 50 mM sodium fluoride). Protein was measured using Bio − Rad/Bradford dye − binding protein micro assay. Western Blot results of 40 μg/lane from three separate experiments were normalized to GAPDH values. For immunoprecipitations, 500 μg of protein was incubated with 5 μg of antibody overnight and 20 μL of EZview Red Protein A Affinity Gel (Sigma) for 4 h prior to washing and SDS-Page.

### Fluorescence microscopy and FACS analysis

1 × 10^5^ cells were grown on 0.17 mm thick coverslips for 5–7 days prior to transfection. Coverslips were fixed and processed as previously described [13]. For zinc imaging, cells were loaded with 5 μM Fluozin-3 (Invitrogen) for 30 min at 37 °C. For FACS analysis using a Becton–Dickinson FACSVerse, non-adherent cells collected by mitotic shake-off and adherent cells harvested by trypsinisation were loaded with 5 μM Fluozin-3 (Invitrogen) for 30 min followed by 30 min recovery in medium. For cell cycle analysis, cells were fixed in 70% ethanol overnight followed by DNA staining with 20 µg/mL propidium iodide (Sigma-Aldrich) plus 0.2 µg/mL DNase − free RNase A and 0.1% Triton X-100 in PBS at 37 ºC for 20 min before FACS analysis and analysed with FlowJo Software version 10 using Watson pragmatic algorithm. Scale bar is 10 μm.

### Proximity ligation assay (PLA)

Cells on eight-well chamber slides (Lab-Tek, Fisher) were fixed followed by PLA using Duolink red kit (Sigma) as described previously [13] and where red fluorescent dots indicate two molecules binding. Dots per cell were determined with ImageTool software (Olink) using at least 12 separate images from at least three different experiments and presented as average values ± standard errors.

### Statistical analysis

Statistical analysis was performed using ANOVA with Post-Hoc Dunnett and Tamhane tests. Significance was assumed with * = *p* < 0.05, *** = *p* < 0.01, ∗  ∗  ∗  = *p* < 0.001. Error bars are standard deviation (SD) with at least 3 different experiments.

## Electronic supplementary material

Below is the link to the electronic supplementary material.

Supplementary file1 (PPTX 2860 kb)
