# Supplementary material for: The ZIP6/ZIP10 heteromer is essential for the zinc-mediated trigger of mitosis
Source: Cell Mol Life Sci. 2020 Aug 14;78(4):1781–98. doi: 10.1007/s00018-020-03616-6 (PMC7904737; doi:10.1007/s00018-020-03616-6)
Supplement: Supplementary file 1 — Supplementary file1 (PPTX 2860 kb) [file 18_2020_3616_MOESM1_ESM.pptx]

## Slide 1
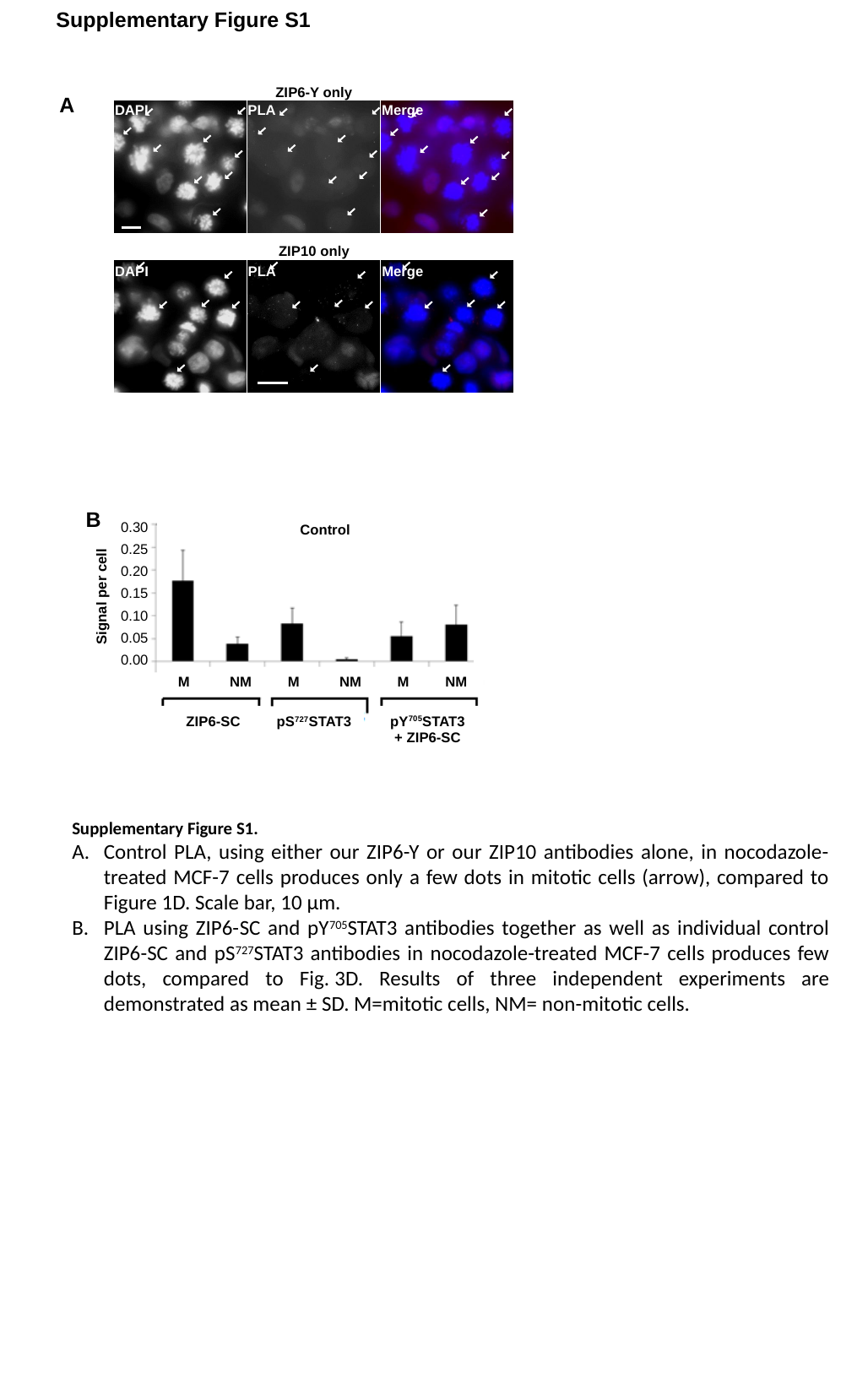

Supplementary Figure S1
ZIP6-Y only
A
Merge
DAPI
PLA
ZIP10 only
Merge
DAPI
PLA
B
0.30
0.25
0.20
0.15
0.10
0.05
0.00
Control
Signal per cell
M NM M NM M NM
ZIP6-SC
pY705STAT3
+ ZIP6-SC
pS727STAT3
Supplementary Figure S1.
Control PLA, using either our ZIP6-Y or our ZIP10 antibodies alone, in nocodazole-treated MCF-7 cells produces only a few dots in mitotic cells (arrow), compared to Figure 1D. Scale bar, 10 μm.
PLA using ZIP6-SC and pY705STAT3 antibodies together as well as individual control ZIP6-SC and pS727STAT3 antibodies in nocodazole-treated MCF-7 cells produces few dots, compared to Fig. 3D. Results of three independent experiments are demonstrated as mean ± SD. M=mitotic cells, NM= non-mitotic cells.

## Slide 2
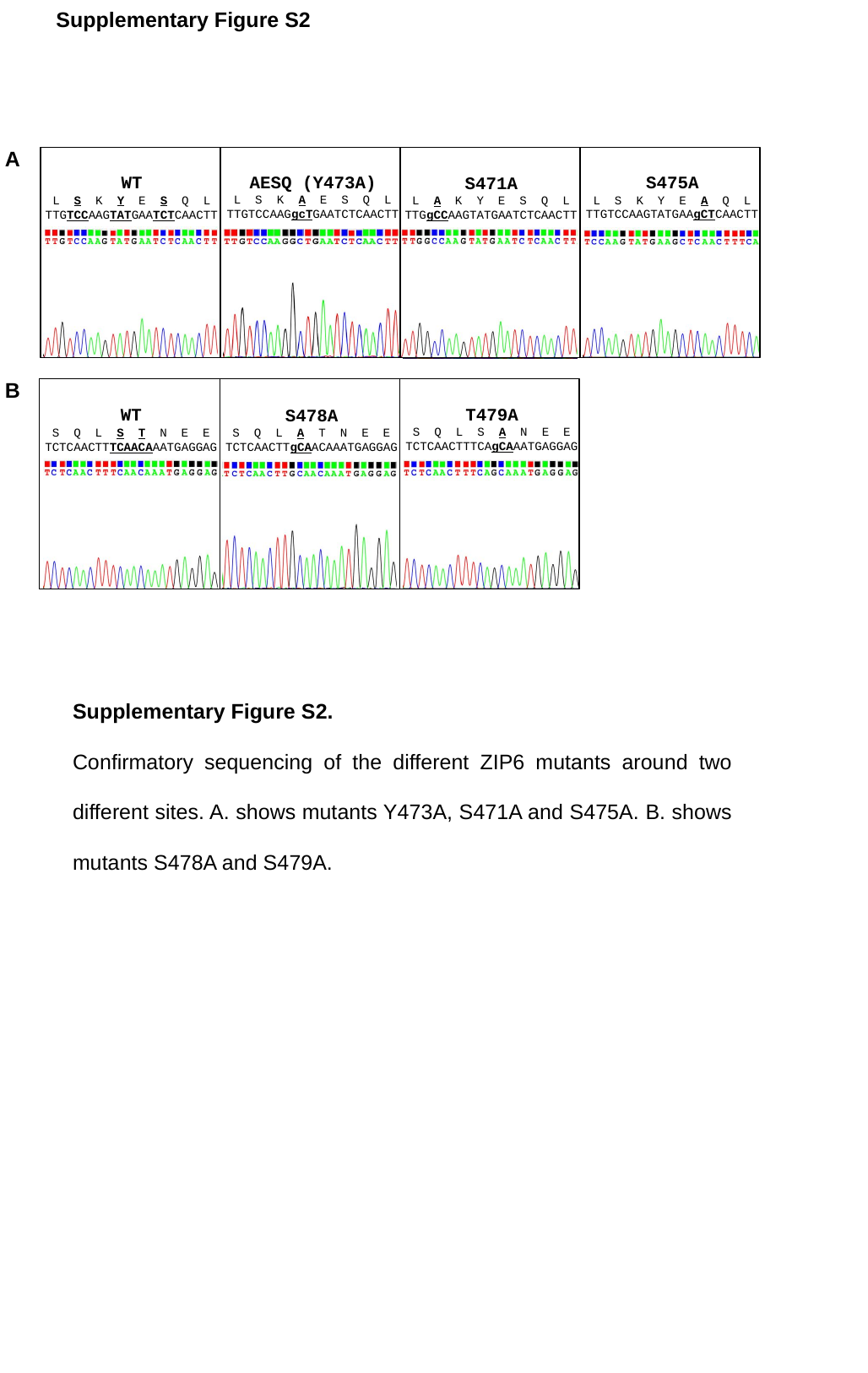

# Supplementary Figure S2
A
AESQ (Y473A)
 L S K A E S Q L
TTGTCCAAGgcTGAATCTCAACTT
S475A
 L S K Y E A Q L
TTGTCCAAGTATGAAgCTCAACTT
WT
 L S K Y E S Q L
TTGTCCAAGTATGAATCTCAACTT
S471A
 L A K Y E S Q L
TTGgCCAAGTATGAATCTCAACTT
B
T479A
 S Q L S A N E E
TCTCAACTTTCAgCAAATGAGGAG
WT
 S Q L S T N E E
TCTCAACTTTCAACAAATGAGGAG
S478A
 S Q L A T N E E
TCTCAACTTgCAACAAATGAGGAG
Supplementary Figure S2.
Confirmatory sequencing of the different ZIP6 mutants around two different sites. A. shows mutants Y473A, S471A and S475A. B. shows mutants S478A and S479A.

## Slide 3
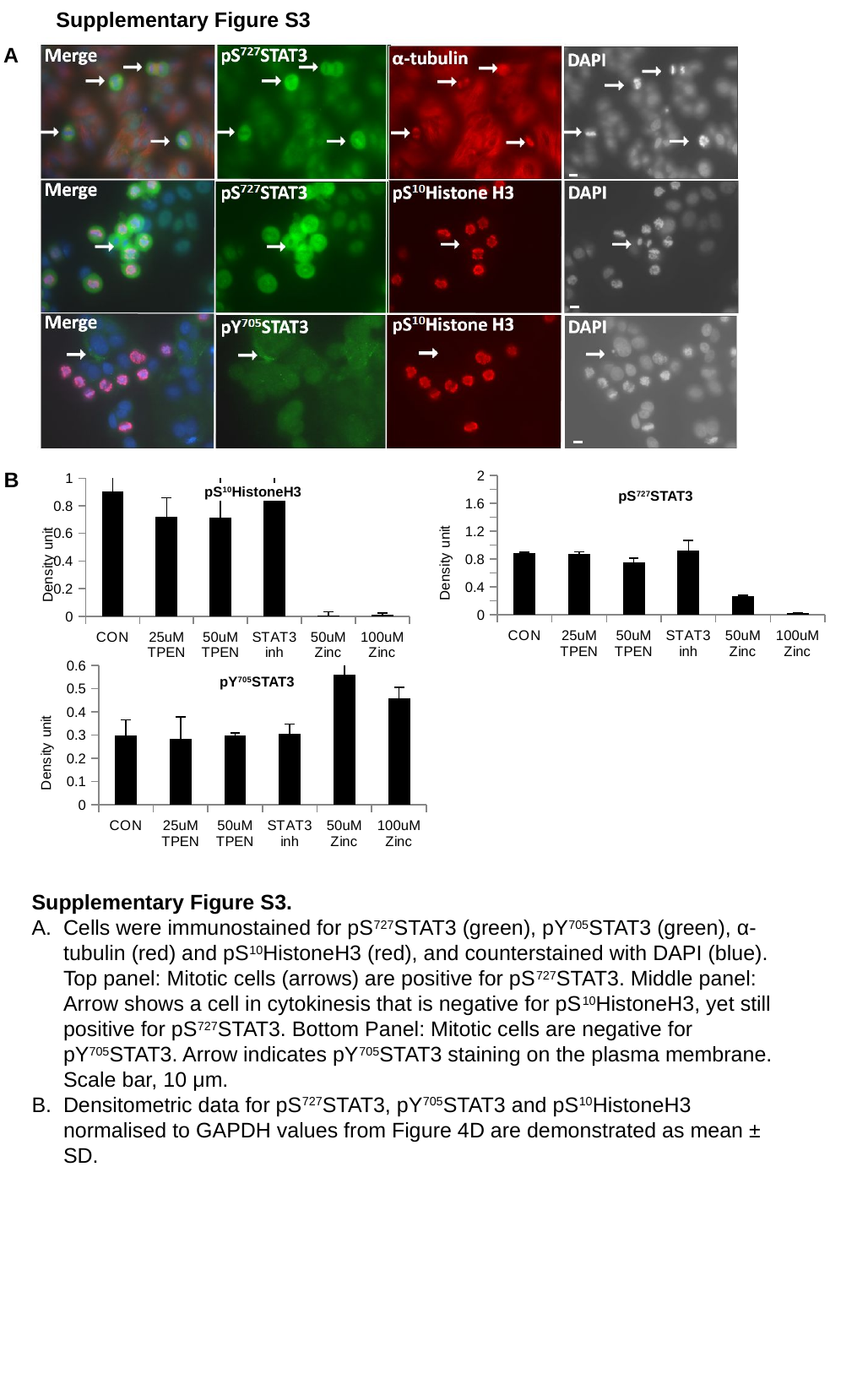

# Supplementary Figure S3
A
Ser727
Ser727
### Chart
| Category | av |
|---|---|
| CON | 0.8863007691182391 |
| 25uM TPEN | 0.8695983159286736 |
| 50uM TPEN | 0.7554149327528764 |
| STAT3 inh | 0.923485740926977 |
| 50uM Zinc | 0.2631202392043594 |
| 100uM Zinc | 0.02250294148207686 |pS727STAT3
B
### Chart
| Category | av |
|---|---|
| CON | 0.9016444018553723 |
| 25uM TPEN | 0.7206940335711692 |
| 50uM TPEN | 0.7160286466398073 |
| STAT3 inh | 0.9265574642022131 |
| 50uM Zinc | 0.005350047436602932 |
| 100uM Zinc | 0.008464973582137875 |pS10HistoneH3
### Chart
| Category | av |
|---|---|
| CON | 0.29821406912664034 |
| 25uM TPEN | 0.28357398950201523 |
| 50uM TPEN | 0.29906170178127417 |
| STAT3 inh | 0.3043956255301434 |
| 50uM Zinc | 0.5600948289019129 |
| 100uM Zinc | 0.45682214760793555 |pY705STAT3
Supplementary Figure S3.
Cells were immunostained for pS727STAT3 (green), pY705STAT3 (green), α-tubulin (red) and pS10HistoneH3 (red), and counterstained with DAPI (blue). Top panel: Mitotic cells (arrows) are positive for pS727STAT3. Middle panel: Arrow shows a cell in cytokinesis that is negative for pS10HistoneH3, yet still positive for pS727STAT3. Bottom Panel: Mitotic cells are negative for pY705STAT3. Arrow indicates pY705STAT3 staining on the plasma membrane. Scale bar, 10 μm.
Densitometric data for pS727STAT3, pY705STAT3 and pS10HistoneH3 normalised to GAPDH values from Figure 4D are demonstrated as mean ± SD.

## Slide 4
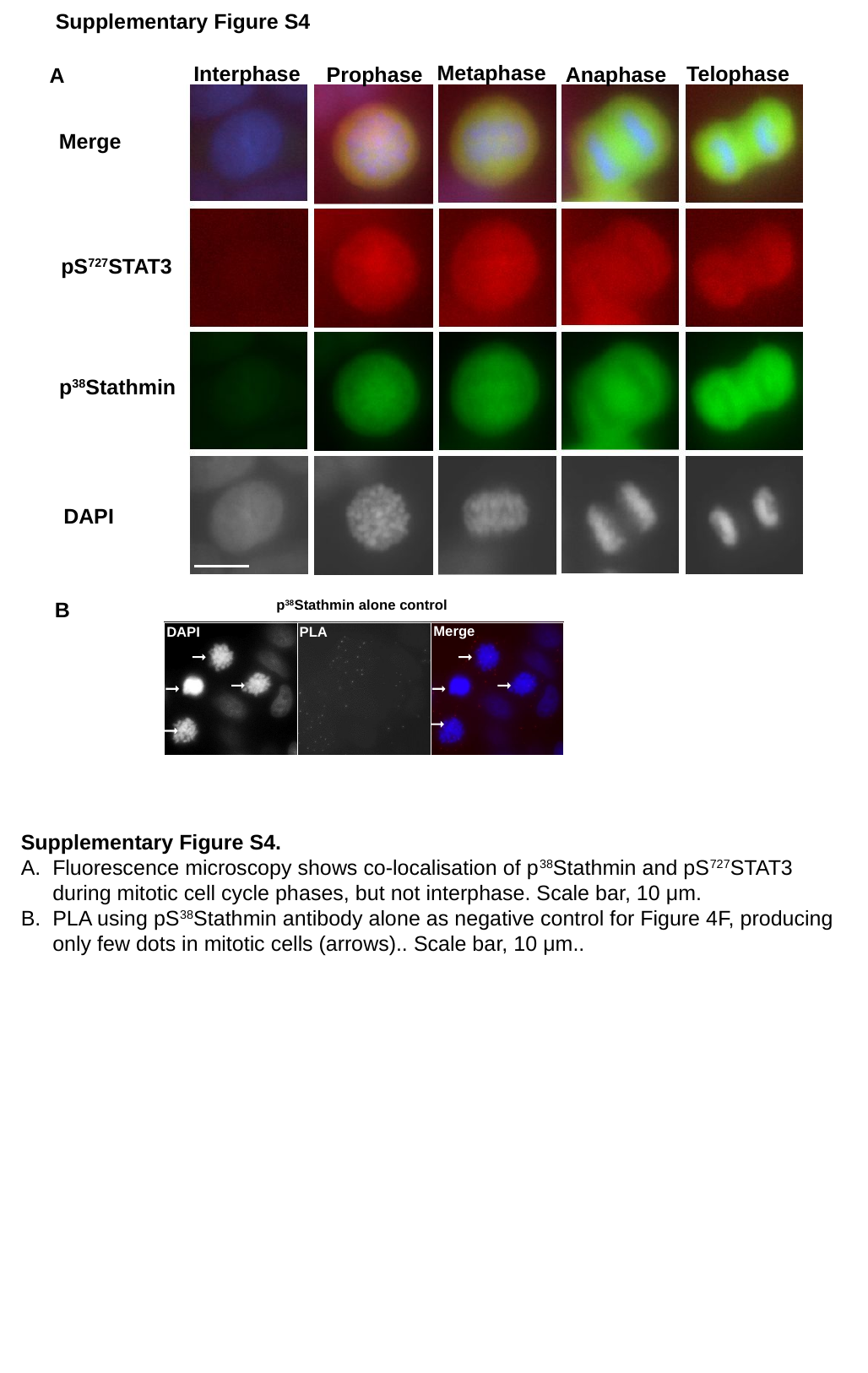

# Supplementary Figure S4
Metaphase
Interphase
Telophase
Prophase
Anaphase
A
Merge
pS727STAT3
p38Stathmin
DAPI
B
p38Stathmin alone control
Merge
DAPI
PLA
pS38Stathmin only
Supplementary Figure S4.
Fluorescence microscopy shows co-localisation of p38Stathmin and pS727STAT3 during mitotic cell cycle phases, but not interphase. Scale bar, 10 μm.
PLA using pS38Stathmin antibody alone as negative control for Figure 4F, producing only few dots in mitotic cells (arrows).. Scale bar, 10 μm..

## Slide 5
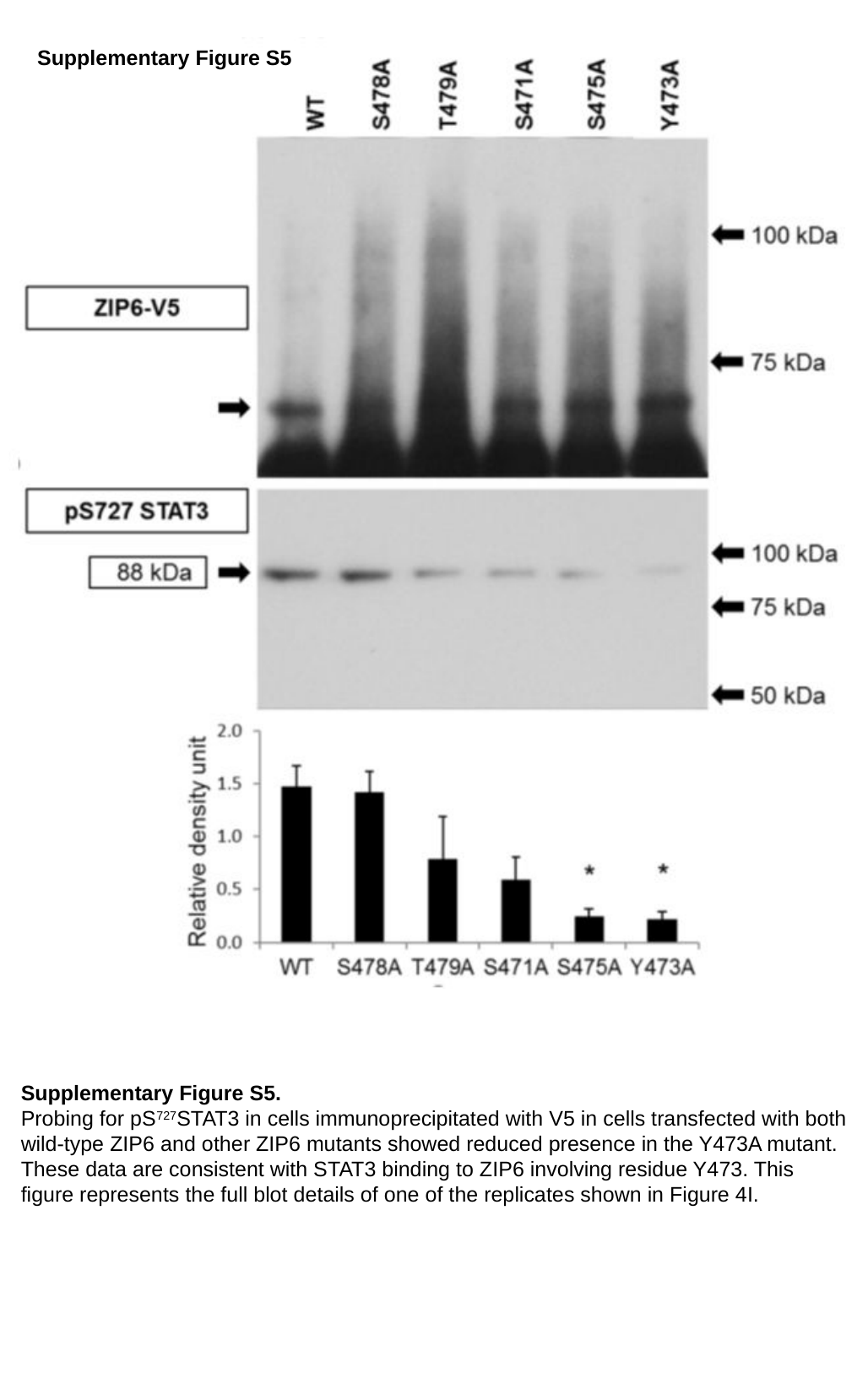

Supplementary Figure S5
Supplementary Figure S5.
Probing for pS727STAT3 in cells immunoprecipitated with V5 in cells transfected with both wild-type ZIP6 and other ZIP6 mutants showed reduced presence in the Y473A mutant. These data are consistent with STAT3 binding to ZIP6 involving residue Y473. This figure represents the full blot details of one of the replicates shown in Figure 4I.
